# A novel personalized time‐varying biomechanical model for estimating lung tumor motion and deformation

**DOI:** 10.1002/mp.18086

**Published:** 2025-09-03

**Authors:** Liang Tan, Wenyou Hu, Liyuan Chen, Huanli Luo, Shi Li, Bin Feng, Xin Yang, Yongzhong Wu, Ying Wang, Fu Jin

**Affiliations:** ^1^ Department of Radiation Oncology Chongqing University Cancer Hospital Chongqing People's Republic of China

**Keywords:** elasticity, finite element analysis, lung biomechanics, patient‐specific modeling, tumor motion and deformation

## Abstract

**Background:**

Accurate prediction of lung tumor motion and deformation (LTMD) is essential for precise radiotherapy. However, existing models often rely on static, population‐based material parameters, overlooking patient‐specific and time‐varying lung biomechanics. Personalized dynamic models that capture temporal changes in lung elasticity are needed to improve LTMD prediction and guide treatment planning more effectively.

**Purpose:**

This study aims to develop a patient‐specific, time‐varying biomechanical model to predict LTMD more accurately.

**Methods:**

Four‐dimensional computed tomography (4DCT) images from 27 patients, each with 10 breathing phases, were analyzed. A finite element model was developed, modeling lung as a hyper‐elastic material and tumor as linear elastic. Lung elasticity parameters, including Young's modulus (*E*) and Poisson's ratio (*v*), were optimized for each phase using Efficient Global Optimization algorithm. Four functions were tested to model the variation of *E* and *v* across different phases. For each patient, average values of these parameters were computed, and their correlation with 11 clinical features was analyzed. The model's accuracy in predicting LTMD was evaluated using tumor center of mass motion error (ΔTCM) and volumetric Dice similarity coefficient (vDSC). Factors influencing the model's accuracy were investigated. Specifically, lung surface traction vector fields (STVFs) were calculated during the transition from end‐expiration to end‐inspiration phases, and their relationship with LTMD was also analyzed.

**Results:**

The first‐order Fourier function provided the best fit among four tested functions, with average R‐squared values of 0.93 ± 0.03 for *E* and 0.91 ± 0.03 for *v*. The average values of *E* and *v* were significantly correlated with patient age. The model showed a mean ΔTCM of 1.47 ± 0.68 mm and a mean vDSC of 0.93 ± 0.02. A negative correlation was found between tumor deformation vDSC and ΔTCM (*r* = −0.55, *p* < 0.05). Higher STVFs were observed near diaphragm and intercostal muscles, with correlations between STVFs and tumor motion amplitude (*r* ≥ 0.92, *p* < 0.05).

**Conclusions:**

These findings offer new insights into developing personalized, time‐varying motion management strategies of lung tumors.

## 1 INTRODUCTION

Lung cancer stands as the leading cause of cancer‐related deaths globally, with 5‐year survival rates for patients varying widely from 4% to 33% across different countries, highlighting a significant disparity in treatment outcomes.^1^, ^2^, ^3^ This variation reflects disparities in early detection, access to care, and treatment precision. Personalized precision treatment is increasingly viewed as essential for improving outcomes. As a method commonly used in treating lung cancer, radiation therapy (RT) is hampered by patient‐specific respiration‐induced lung tumor motion and deformation (LTMD).^4^, ^5^, ^6^, ^7^ These motions complicate tumor localization, which disrupts radiation dose distribution, reduces treatment efficacy, and increases the risk of adverse effects.^8^, ^9^, ^10^

To address these challenges, various techniques such as image reconstruction, registration, and motion management have been developed.^11^, ^12^, ^13^, ^14^, ^15^ Among these, finite element analysis‐based image registration (FEAIR) has emerged as a promising approach.^16^, ^17^ By incorporating patient‐specific anatomical and physiological data, FEAIR‐based lung biomechanical models can predict lung and tumor behavior more accurately, offering a personalized approach to LTMD simulation.

Most previous studies have focused on two key aspects of lung biomechanical modeling: boundary conditions and material properties. Accurate loading conditions, driven by displacement fields as boundary conditions,^16^, ^17^, ^18^ are essential for simulating lung deformation. However, the main challenge lies in capturing the dynamic elasticity of lung tissue.

While models likes Mooney‐Rivlin,^19^ Neo‐Hookean (NH),^17^ Saint‐Venant‐Kirchhoff (SVK),^18^, ^20^, ^21^ and Yeoh^16^, ^22^ address lung nonlinearity, they fail to fully incorporate patient‐specific characteristics, limiting their accuracy in simulating the lung's deformation during respiration.

Lung tissue elasticity is highly variable across individuals.^23^, ^24^, ^25^ Key bi‐parameters include Young's modulus (*E*) and Poisson's ratio (*v*), which define lung mechanics, range from 0.10 kPa to 7.8 kPa and 0.1 to 0.49, respectively. This variability, along with dynamic elasticity changes over time during respiration,^26^ complicates the development of accurate models. Additionally, direct measurement of lung elasticity remains costly and technically challenging, restricting the personalization of biomechanical models. Although indirect optimization methods, such as minimizing displacement discrepancies or maximizing geometric similarities,^16^, ^19^, ^23^, ^27^ have been explored, they do not fully account for temporal dynamics and individual variability of lung properties.

In this paper, we developed a novel personalized time‐varying bi‐parametric hyper‐elastic lung biomechanical model to estimate LTMD. Unlike prior approaches that rely on static or population‐averaged elasticity parameters, our model dynamically optimizes patient‐specific *E* and *v* across the respiratory cycle using an Efficient Global Optimization (EGO) algorithm.^28^ Based on lung volume change, we adaptively select between two hyper‐elastic material models (SVK or NH), enabling physiological flexibility across patients and breathing patterns.

Beyond motion prediction, we further investigate the temporal evolution of lung elasticity and identify patient‐level features that influence these changes through regression analysis. Lastly, we characterize the stress transfer from lung to tumor using 3D stress–strain analysis, offering new insights into lung–tumor biomechanical coupling. These contributions support a more individualized, time‐adaptive motion management framework, with potential clinical utility in radiotherapy planning, adaptive margin design, and real‐time tracking.

## 2 METHODS AND MATERIALS

**FIGURE 1:**
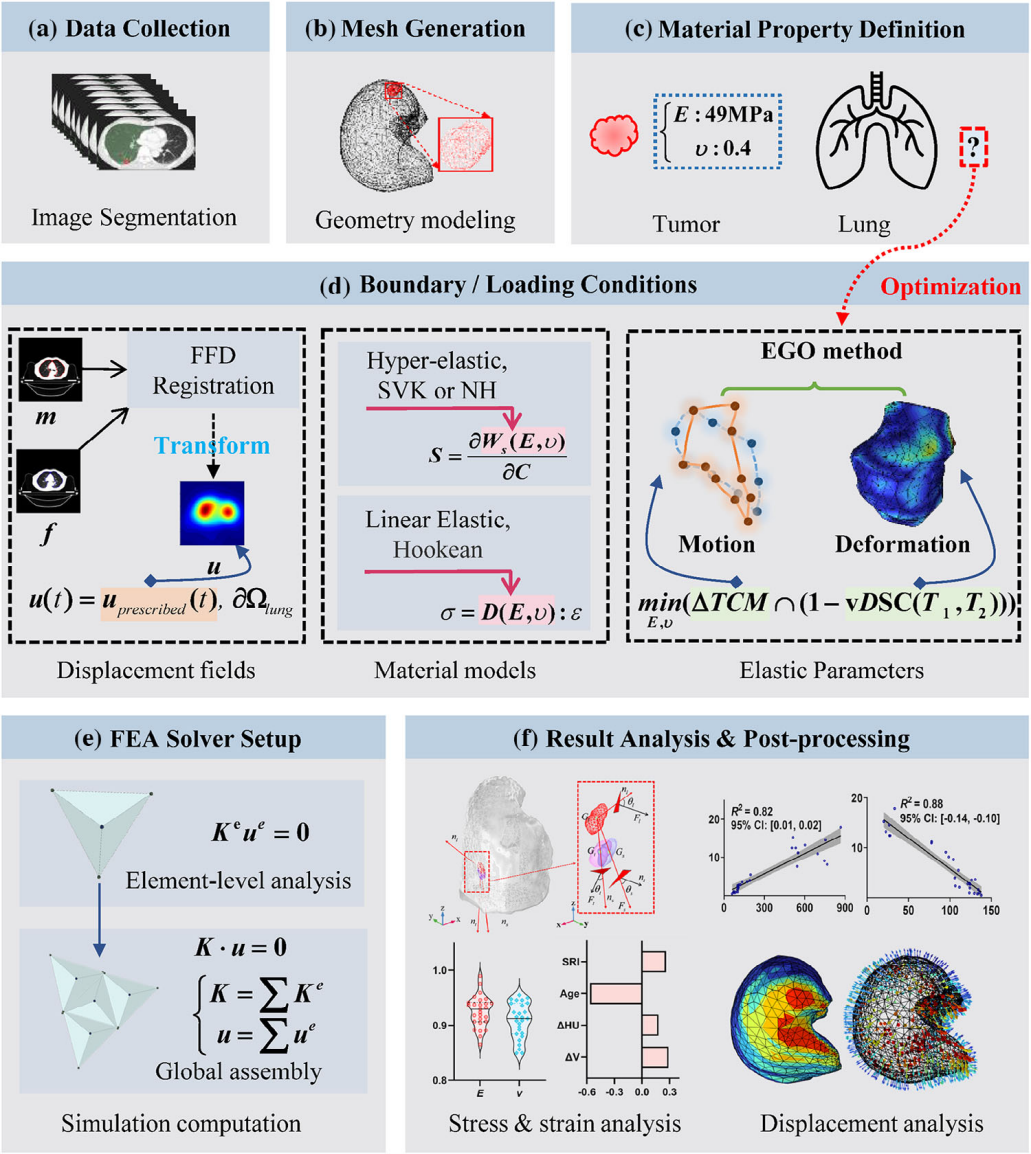
The workflow for developing and analyzing lung biomechanical model. ***E***, Young's modulus; ***v***, Poisson's ratio; *m*, moving image; *f*, fixed image; ***u***, displacement field; FFD, free‐form deformation; SVK, Saint‐Venant‐Kirchhoff; NH, Neo‐Hookean; EGO, Efficient Global Optimization; ΔTCM, tumor's center of Mass motion error; vDSC, volumetric Dice similarity coefficient; FEA, finite element analysis; ***K***, Stiffness Matrix; *e*, element‐level.

The workflow for constructing the FEAIR‐based lung biomechanical model is shown in Figure 1, detailing key steps such as geometry construction, material properties assignment, boundary conditions application, and result post‐processing.

### 2.1 Datasets

This study used four‐dimensional computed tomography (4DCT) scans from 27 lung cancer patients at Chongqing University Cancer Hospital, with ethical approval from the hospital's ethics committee (NO. CZLS2023164‐A). Each scan included 10 respiratory phases, sorted by Varian Respiratory Gating System™ (RPM, Version 1.7, Varian Medical System.Inc., Palo Alto, CA). Image pixel sizes ranged from 0.98 × 0.98 mm to 1.37 × 1.37 mm. All patients underwent 4DCT scanning under free‐breathing conditions. To reduce artifacts caused by irregular respiration, several quality control measures were applied, including pre‐scan breathing training to encourage stable respiratory patterns, manual inspection of image sequences, and validation of phase sorting based on anatomical continuity. Additional patient details are provided in Table 1.

**TABLE 1:** Summary of patient and lung/tumor characteristics.

Group	Characteristics	Mean (range) or Number
Patient	Age (year)	64 (41–85)
	Respiration cycle (second)	4.0 (3.9–4.9)
	BMI^a^ (kg/m^2^)	21.8 (16.8–28.7)
	Female	9
	Male	18
Lung	Motion amplitude^b^ (mm)	20.3 (7.7–35.1)
	Volume change (%)	13 (7–39)
Tumor	Motion amplitude^b^ (mm)	6.3 (0.5–17.9)
	Location (left lung)	12
	Location (right lung)	15
	Size (cm^3^)	5.7 (0.4–22.38)

^a^ Body Mass Index.

^b^ Motion amplitude refers to the maximum centroid displacement during the respiratory cycle, calculated as $\sqrt{{\mathrm{SI}}^{2}+{\mathrm{AP}}^{2}+{\mathrm{RL}}^{2}}$, where SI, AP, and RL represent the superior–inferior, anterior–posterior, and right–left directions, respectively.

### 2.2 Data preprocessing

Lung masks for each respiratory phase were delineated using the deep learning‐based automated algorithm^29^ and subsequently reviewed by a radiation oncologist, with necessary edits made. The oncologist adjusted lung segmentations to correct motion‐induced distortions, such as boundary misalignments and partial volume effects. The Gross Tumor Volume (GTV) for the lung tumors was obtained from the clinical radiation therapy planning target volumes. To ensure data fidelity, we maintained consistent voxel spacing and spatial orientation at each step, and verified that all exported STL models were watertight and free of mesh defects. Additionally, we quantitatively compared the surface area and volume of the STL models with the original segmentation masks for all 10 respiratory phases. The observed geometric discrepancies were minimal (all < 2%), additional details are provided in Table S1.

### 2.3 Lung biomechanics modeling

The lung and tumor geometries were obtained from segmented 4DCT images and discretized into free tetrahedral meshes. Tetrahedral meshes were automatically generated using COMSOL's built‐in free meshing algorithm. To assess mesh sensitivity, we compared lung and tumor geometries under different mesh resolutions. Variations in surface area and volume were negligible (< 0.5%), and a convergence test showed displacement results stabilized at intermediate mesh densities. We therefore adopted the Normal mesh size, balancing computational efficiency and accuracy. Detailed comparisons are provided in the Supplementary Material Figure S1.

In this study, the lung and tumor were modeled as a homogeneous and isotropic material within each respiratory phase. The lung was modeled as a hyper‐elastic material, with *E* and *v* optimized using the EGO method (a gradient‐free optimization approach that uses a surrogate model [Gaussian Process]).^28^, ^30^ The tumor was modeled as a linear elastic material, with *E* set at 49 MPa and *v* of 0.4^18^. Lung displacement fields obtained through free‐form deformation (FFD) image registration,^31^ were applied as Dirichlet boundary conditions. The accuracy of FFD‐based registration within the lung (excluding the tumor region) was verified and confirmed by experienced clinicians. Finite element analysis (FEA) was performed in COMSOL using transient analysis over each patient's personalized respiratory cycle obtained during data acquisition. A new mesh was generated for each phase, with surface displacement as the initial condition. The parallel sparse direct solver (PARDISO) was employed for FEA. Stress‐strain and displacement analyses were performed to evaluate lung‐tumor interactions and deformation.

For modeling the lung's nonlinear behavior, two hyper‐elastic material models were employed,^32^ with the choice of model based on the volume change rate (*J*) between the end‐expiration (*P*_50_) and end‐inspiration (*P*_00_) phases, defined as *J* = *V*(*P*_00_) / *V*(*P*_50_). Specifically, the SVK model was applied for *J* ≤ 1.1, while the NH model was used for *J* > 1.1, based on the deformation ranges suitable for each model.^33^, ^34^ The stress‐strain relationship for both models is described by the strain energy density function (*W*):

(1) $$W=\left\{\begin{array}{c} \frac{\lambda }{2}I_{1}^{2}+\mu \left(I_{1}^{2}-2I_{2}^{2}\right),\mathrm{SVK}\\ \frac{\mu }{2}\left(I_{1}-3\right)-\mu \ln\left(J\right)+\frac{\lambda }{2}\ln{\left(J\right)}^{2},\mathrm{NH} \end{array}\right.$$

where *I*_1_ and *I*_2_ denote the invariants of the deviatoric right Cauchy‐Green deformation tensor, and *λ* and *μ* are the Lamé parameters, related to *E* and *v* as shown below:

(2) $$\lambda =\frac{vE}{\left(1+v)(1-2v\right)},\mu =\frac{E}{2(1+E)}.$$

To simulate tumor motion throughout the respiratory cycle, we modeled the lung and tumor as a coupled system. No external forces were applied directly to the tumor; instead, the tumor's motion was driven entirely by the displacement field of the surrounding lung tissue. The gross tumor volume (GTV) was propagated across the ten respiratory phases by sequentially applying inter‐phase deformation fields in the following order: $P_{50}\to P_{60}\to P_{70}\to P_{80}\to P_{90}\to P_{00}\to P_{10}\to P_{20}\to P_{30}\to P_{40}\to P_{50}$, forming a complete respiratory cycle.

### 2.4 Model validation

Model validation was conducted by comparing simulated LTMD with observed data using two key metrics: tumor's center of mass (TCM) motion error and volumetric Dice Similarity Coefficient (vDSC). To ensure robustness, validation was performed across tumors with a wide range of volumes (0.4–22.38 cm^3^), as shown in Table 1. The TCM motion error (ΔTCM) was computed as the Euclidean distance between simulated and observed TCM positions, while vDSC measured the overlap between the simulated (*V*_sim_) and observed (*V*_obs_) tumor volumes. The mathematical definitions of both metrics are as follows:

(3) $$\begin{array}{cc} & \Delta \mathrm{TCM}\\ & =\sqrt{{\left(x_{\mathrm{sim}}-x_{\mathrm{obs}}\right)}^{2}+{\left(y_{\mathrm{sim}}-y_{\mathrm{obs}}\right)}^{2}+{\left(z_{\mathrm{sim}}-z_{\mathrm{obs}}\right)}^{2}}, \end{array}$$

(4) $$\mathrm{vDSC}=\frac{2(V_{\mathrm{sim}}\cap V_{\mathrm{obs}})}{V_{\mathrm{sim}}+V_{\mathrm{obs}}},$$

where (*x*_sim_, *y*_sim,_ *z*_sim_) are the coordinates of the simulated TCM, and (*x*_obs_, *y*_obs,_ *z*_obs_) are the coordinates of the observed TCM. (*V*_sim_∩*V*_obs_) is the volume of the intersection between *V*_sim_ and *V*_obs_.

### 2.5 Optimization of lung elasticity parameters

To optimize the lung's elastic parameters (*E* and *v*), the EGO algorithm was employed. The objective function was defined as a weighted combination of ΔTCM and vDSC,^16^ with equal weights of 0.5. The vDSC component was treated as a maximization goal. The combined optimization problem can be formulated as:

(5) $${\mathrm{Objective}}_{\left(P_{i},P_{j}\right)}^{n}=\Delta \mathrm{TCM}(P_{i},P_{j})+(1-\mathrm{vDSC}(P_{i},P_{j})),$$

where *n* represents the 𝑛‐th patient, with *n* ranging from 1 to 27, and *P*_i_ and *P*_j_ are respiratory phases from 4DCT images, where i ≠ j. The goal is to minimize this sum to achieve the best match between the simulated and observed LTMD.

The optimization variables *E* and *v* were constrained within the ranges [2, 11] kPa and [0.1, 0.49] ^23^, respectively. The algorithm ran with a convergence tolerance of 0.01, a maximum of 500 iterations, and a surrogate model resolution tolerance of 1E−16.

### 2.6 Data postprocessing

As there is limited literature on the dynamic changes of lung elasticity parameters, this study represents an initial exploratory attempt. To model the approximate periodicity of lung elasticity during respiration, four different models were tested: including exponential function (Exp2), Fourier function (Fourier1), Gaussian function (Gauss2), and quadratic function (Poly2). To avoid overfitting, we selected low‐complexity curve models (2–4 parameters) and fit them only within the observed respiratory phases. The number of terms for each model is 2, 1, 2, and 2, respectively, as shown below.

(6) $$f\left(t\right)=\left\{\begin{array}{c} c_{1}\cdot e^{c_{2}t}+c_{3}\cdot e^{c_{4}t},\mathrm{Exp}2\\ c_{1}\cdot \cos\left(c_{2}\cdot t\right)+c_{3}\cdot \sin\left(c_{4}\cdot t\right)+c_{5},\mathrm{Fourier}1\\ c_{1}\cdot e^{-({\left(t-c_{2}\right)/c_{3})}^{2}}+c_{4}\cdot e^{-({\left(t-c_{5}\right)/c_{6})}^{2}},\mathrm{Guass}2\\ c_{1}\cdot t^{2}+c_{2}\cdot t+c_{3},\mathrm{Poly}2 \end{array}\right..$$

In this equation, *c*_i_ (i = 1, 2, …, 6) are coefficients of the functions, *t* represents time, and f(*t*) represents the time‐varying lung Young's modulus and Poisson's ratio.

Furthermore, we analyzed the correlation between patient physiological and imaging features (11 in total) and averaged lung elasticity parameters. Feature selection and dimensionality reduction were performed using the Least Absolute Shrinkage and Selection Operator (LASSO) method, followed by LASSO regression to assess relationship with the average lung elasticity. Given the limited sample size (27 cases), Leave‐One‐Out Cross‐Validation was applied in both steps to optimize the model and ensure robustness. The 11 features used in the regression analysis are summarized in Table 2.

**TABLE 2:** Summary of extracted features.

Feature	Formula / Definition	Description/ Abbreviation
*X*_1_	$\left(V_{00}-V_{50}\right)/V_{50}$ ^a^	Lung volume (ΔV)
*X*_2_	$\left(HU_{00}-HU_{50}\right)/HU_{50}$ ^b^	Average CT value (ΔHU)
*X*_3_	$\left(x_{00}-x_{50}\right)/x_{50}$ ^c^	Lung boundary length (Δx)
*X*_4_	$\left(y_{00}-y_{50}\right)/y_{50}$	Lung boundary length (Δy)
*X*_5_	$\left(z_{00}-z_{50}\right)/z_{50}$	Lung boundary length (Δz)
*X*_6_	$\left(S_{00}/V_{00}-S_{50}/V_{50}\right)/\left(S_{50}/V_{50}\right)$	Surface area‐to‐volume ratio (ΔS/V)
*X*_7_	—	Patient age (Age)
*X*_8_	—	Lung motion amplitude (LMA)
*X*_9_	weight (kg) / height (m)^2^	Body mass index (BMI)
*X*_10_	$\sqrt{{\left(\left(\lambda_{1}-\lambda_{2}\right)/\lambda_{2}\right)}^{2}+{\left(\left(\lambda_{2}-\lambda_{3}\right)/\lambda_{3}\right)}^{2}}$ ^d^	Anisotropic deformation index (ADI)^35^
*X*_11_	$\left(2/\pi \right)\cdot \arctan\left(\frac{\lambda_{3}\left(\lambda_{1}-\lambda_{2}\right)}{\lambda_{2}\left(\lambda_{2}-\lambda_{3}\right)}\right)$	Slab‐rod index (SRI)

^a^ Subscripts “00” and “50” denote respiratory phases $P_{00}$ and $P_{50}$, respectively.

^b^ CT value in Hounsfield Units (HU), averaged over the lung region.

^c^ Boundary lengths derived from bounding box along x,y,z axes.

^d^ $\lambda_{1}>\lambda_{2}>\lambda_{3}$ are principal strains (eigenvalues of deformation tensor).

### 2.7 Lung surface traction vector fields analysis

**FIGURE 2:**
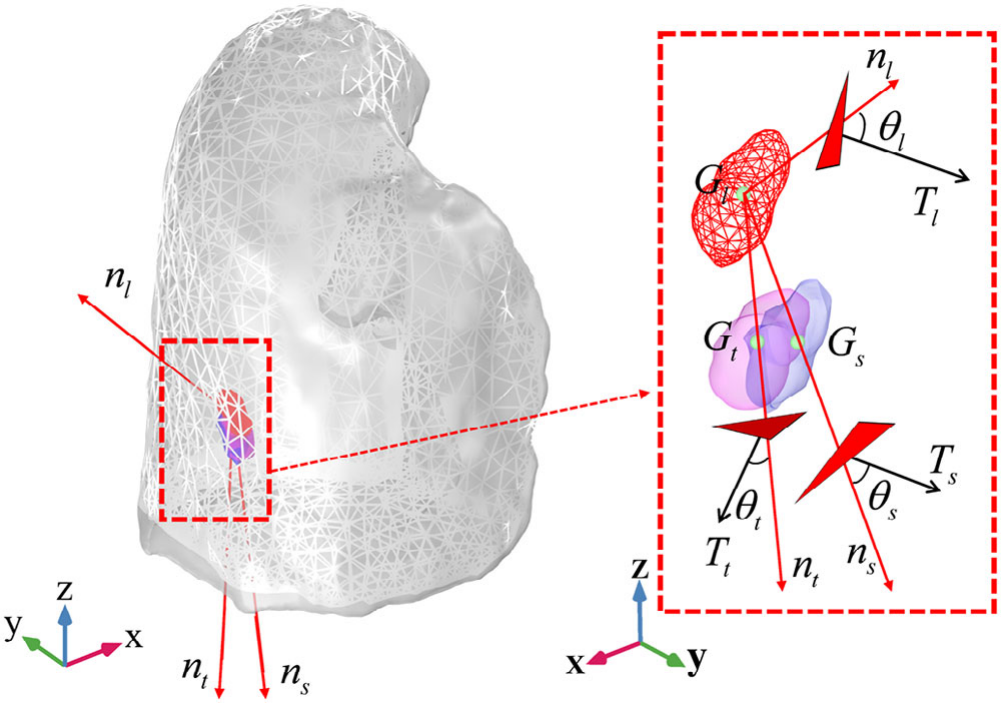
Illustration of local lung surface traction vector fields (STVFs) and key parameters for tumor motion analysis. Explanation of variables: *G*, gravity of tumor; *n*, normal vector; *T*, traction force; *θ*, angle between *T* and *n*. The subscript *t*, *s* and *l* distinguish three different directions: *t*, from the *G* of end‐expiration phase (*G*_50_) to the true *G* of end‐inspiration phase (*G*_00_); *s*, from the *G*_50_ to simulate *G*_00_; *l*, from the *G*_50_ to at the lung surface closest to the *G*_50_.

The lung surface traction vector fields (STVFs) were computed to examine the spatial distribution of traction forces. These forces (*T*) are described by a vector field indicating both magnitude and direction, and serve as boundary load vectors linking the deformed and undeformed states. For additional theoretical details, readers are referred to the COMSOL Multiphysics documentation (https://doc.comsol.com/6.3/docserver/#!/com.comsol.help.sme/sme_ug_theory.06.024.html). The definition of *T* is expressed as follows:

(7) $$\left\{\begin{array}{c} T=Pn_{0}\\ \sigma =J^{-1}PF^{T},\det\left(F\right) \end{array}\right.,$$

where *P* is the first Piola‐Kirchhoff stress tensor, *n*_0_ is the normal vector, σ is the Cauchy stress, *J* is the Jacobian matrix, and *F* the deformation gradient. The role of STVFs in LTMD was analyzed by considering three local STVFs, with detailed annotations provided in Figure 2.

### 2.8 Implementation

Image registration were performed using the Insight Toolkit (ITK) in C++. The biomechanical model was conducted with COMSOL Multiphysics numerical analysis Software (Version 6.2), while lung elastic parameters optimization was implemented in MATLAB (MathWorks, USA). LASSO regression and additional modeling tasks were carried out using PyCharm Professional Edition (Version 2022.2.2).

## 3 RESULTS

### 3.1 Optimization of elasticity parameters

**FIGURE 3:**
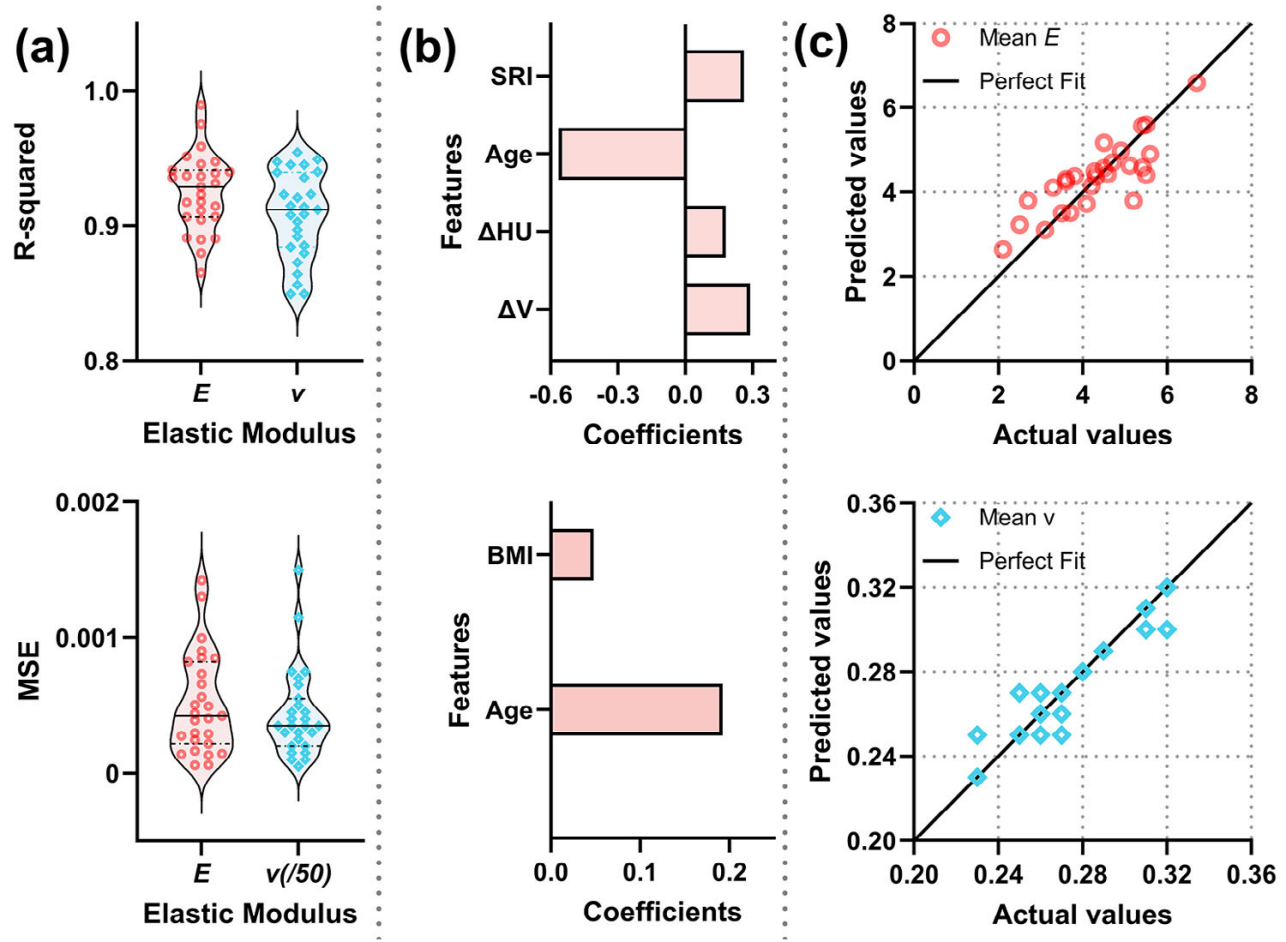
Post‐processing results of elasticity parameters. (a) Fitting performance of the four functions; (b) and (c) Regression coefficients and predictive accuracy of the averaged lung Young's modulus and Poisson's ratio using lasso regression, respectively. (c) Comparison of biomechanically optimized (“actual”) and regression‐estimated (“predicted”) values of Young's modulus and Poisson's ratio. The actual values are obtained from the subject‐specific optimization process, while the predicted values are estimated using LASSO regression based on patient features. Young's modulus (*E*); Poisson's ratio (*v*); exponential function (Exp2); Fourier function (Fourier1); Gaussian function (Gauss2); quadratic function (Poly2); mean‐square error (MSE); slab‐rod index (SRI); body mass index (BMI); relative change in volume (ΔV); relative change in the average CT value (ΔHU).

Figure 3(a) shows the fitting performance of Exp2, Fourier1, Gauss2, and Poly2 for lung elasticity parameters (*E* and *v*), with an R^2^ greater than 0.70 and a mean squared error (MSE) of less than 0.003 for all patients. Detailed results across all patients are provided in Tables S2 and S3, Figures S2 and S3. Fourier1 and Gauss2 showed the highest median R^2^ values for both *E* and *v*, while Gauss2 showed greater variability in MSE. Figure 3(b) presents the coefficients of the Lasso regression model, showing that the averaged Young's modulus and Poisson's ratio were significantly associated with age, with coefficients of ‐0.56 and 0.2, respectively. Figure 3(c) depicts the relationship between the actual/observed (mean) and predicted values of the averaged *E* and *v*, with MSE of 0.52 and 0.0023, respectively.

### 3.2 Model performance

**FIGURE 4:**
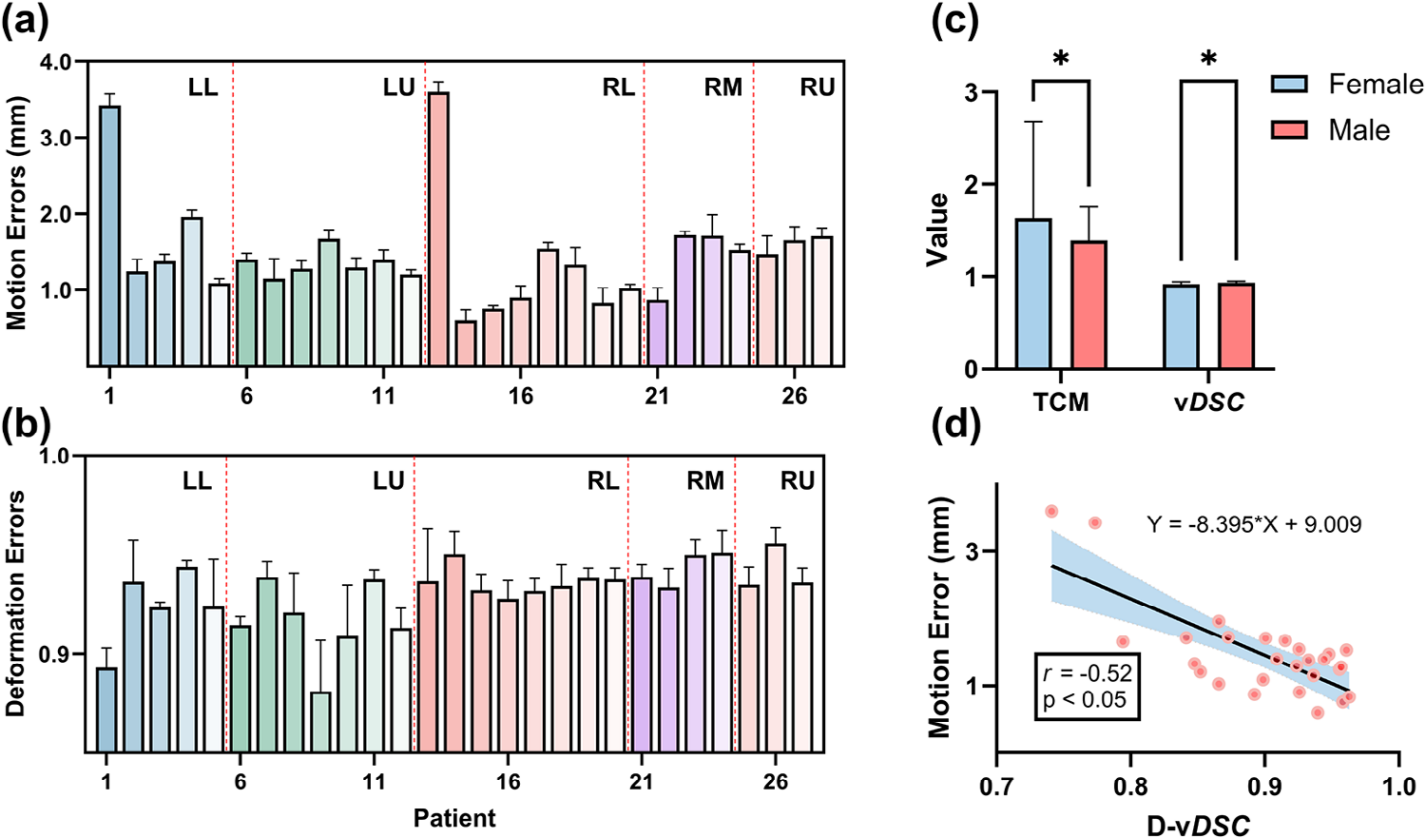
Model performance in predicting tumor motion and deformation. (a) and (b) Tumor motion and deformation errors across lung lobes: left lower (LL), left upper (LU), right lower (RL), right middle (RM), and right upper (RU) lobes; (c) Comparison of TCM motion errors and volumetric dice similarity coefficients (vDSC) between female and male patients; (d) Correlation between the tumor deformation vDSC (D‐vDSC) and TCM motion errors.

Figure 4(a) and (b) demonstrate the performance of the lung biomechanical model in predicting LTMD at different locations, including the left lower lobe (LL), left upper lobe (LU), right lower lobe (RL), right middle lobe (RM), and right upper lobe (RU). The model achieved an overall mean TCM motion error of 1.47 ± 0.68 mm and a mean vDSC of 0.93 ± 0.02. The TCM motion errors for each lobe were 1.82 ± 0.87 mm (LL), 1.34 ± 0.21 mm (LU), 1.32 ± 0.92 mm (RL), 1.46 ± 0.39 mm (RM), 1.61 ± 0.20 mm (RU). Similarly, the vDSC values were 0.92 ± 0.02 (LL), 0.91 ± 0.02 (LU), 0.94 ± 0.01 (RL), 0.94 ± 0.01 (RM), 0.94 ± 0.01 (RU). Significant differences in TCM motion error and vDSC were observed between female and male patients (Figure 4(c)). Additionally, Figure 4(d) illustrates a significant negative correlation between D‐vDSC and simulated TCM motion errors (*r* = ‐0.52, *p* < 0.05).

In addition, we analyzed the relationship between the model's motion prediction errors and the true tumor motion amplitudes. Detailed results are provided in Table S4 of the Supplementary Material. In the low‐amplitude group (≤1 mm), prediction errors remained consistently low, all below 0.7 mm. For moderate amplitudes (1–10 mm), errors generally increased with amplitude, ranging from 0.72 mm to 2.42 mm. In the high‐amplitude group (> 10 mm), prediction errors were relatively stable, mostly between 1.15 mm and 2.18 mm, with one notable outlier (case27, 17.88 mm) exhibiting a larger error of 3.60 mm, which may be attributed to substantial tumor deformation (D‐vDSC = 0.74) under extreme motion.

### 3.3 Lung surface traction forces

**FIGURE 5:**
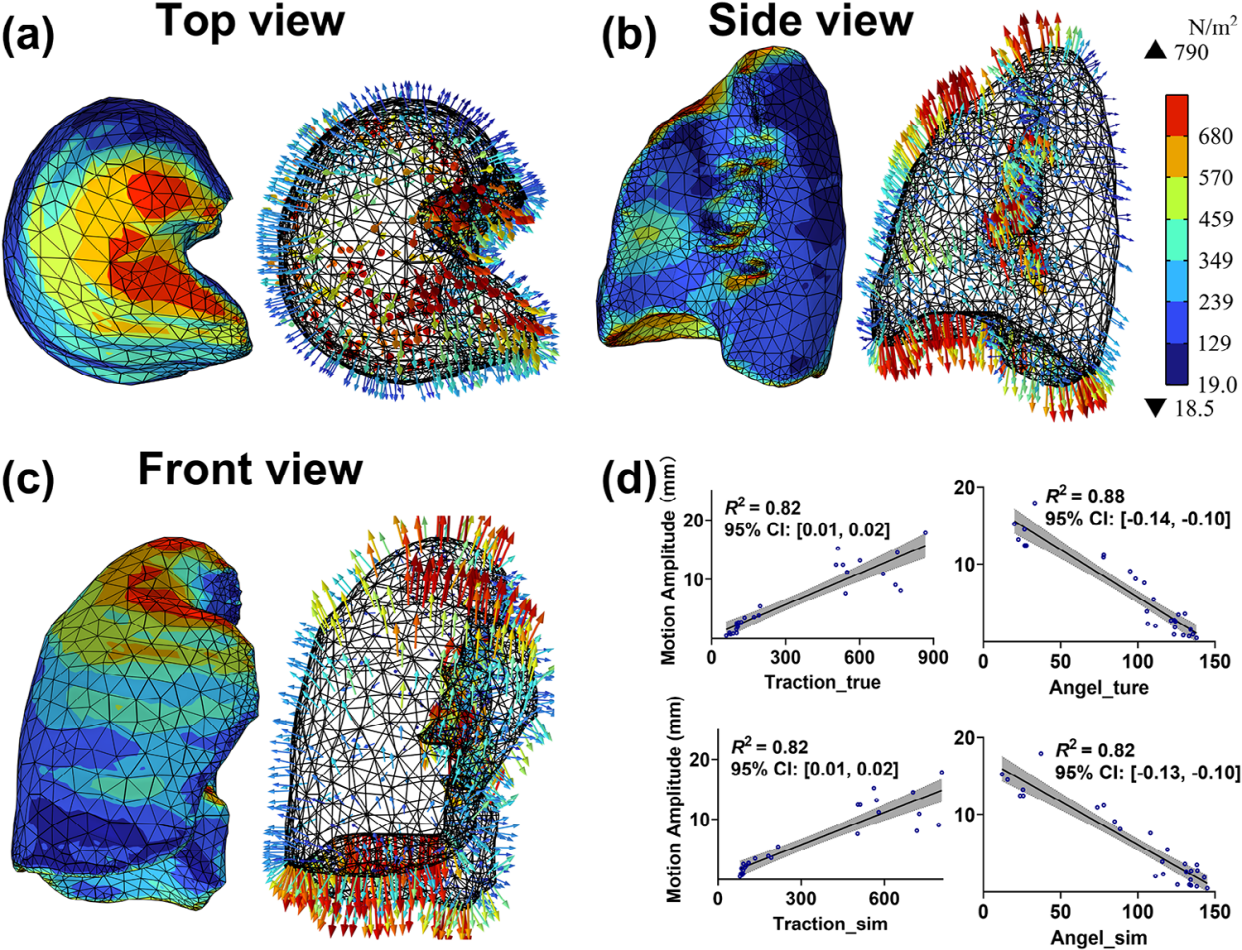
Lung surface traction forces and their correlation with tumor motion amplitude. (a), (b), and (c) Lung surface traction vector fields during the respiratory cycle (from end‐expiration to end‐inspiration), shown in axial, sagittal and coronal views, respectively; (d) Correlation between traction force amplitude/angle and tumor motion amplitude.

Figure 5(a), (b), and (c) show an example of lung surface traction vector fields from the end‐expiration to end‐inspiration phases, presented in axial, sagittal and coronal views, respectively. These views reveal higher traction forces at the lung apex, anterior surface, and bronchi, and lung base in regions adjacent to the diaphragm. Notably, an elongated region of high traction on the anterior lung surface corresponds to the intercostal muscles. Figure 5(d) presents the correlation between the amplitude and direction of traction forces in specific directions and lung tumor motion amplitude. The results show strong correlations: true traction amplitude (*r* = 0.85), true traction angle (*r *= ‐0.84), simulate traction amplitude (*r* = 0.86), simulate traction angle (*r* = ‐0.80), with all *p*‐values less than 0.001. Additionally, the goodness of fit, measured by *R* ^2^, was greater than 0.82 for the linear regression.

## 4 DISCUSSION

### 4.1 Performance of the biomechanical model

The proposed lung biomechanical model demonstrated high accuracy in predicting LTMD across different lung lobes, with an overall mean TCM motion error of 1.47 ± 0.68 mm and vDSC of 0.93 ± 0.02. The TCM motion errors ranged from 1.32 ± 0.92 mm to 1.82 ± 0.87 mm, and consistently high vDSC values between 0.91and 0.94 (Figure 4(a), (b)). Notably, although the mean TCM error (∼1.5 mm) is close to the spatial resolution of 4DCT, it remains physically plausible. This is because the tumor centroid was computed from reconstructed 3D meshes, allowing for sub‐voxel accuracy and consistent tracking across phases. The model incorporated patient‐specific, time‐varying elasticity parameters to induce a hyper‐elastic biomechanical model based on image registration. Depending on the volume changes between the end‐expiration and end‐inspiration phases, either the SVK or NH model was selected. Research has shown that the hybrid biomechanical model and image registration method help correct registration biases caused by nonlinear motion or image errors,^17^ significantly improving the accuracy of lung tumor motion and deformation predictions.

When comparing to previous research, our results show superior performance. Tehrani et al. reported accuracies of 2.31 ± 0.72 mm and 0.90 ± 0.03 for TCM motion error and vDSC using a NH material model, and 2.26 ± 0.74 mm and 0.90 ± 0.03 using the Mooney‐Rivlin material.^19^ Jafari et al. similarly reported a mean vDSC of 0.85 ± 0.06 using a Yeoh hyper‐elastic model on 5 subjects.^16^ The discrepancies in these results are likely attributable to the differences in the material models and model complexities. Our model benefits from patient‐specific elasticity parameters, which may better capture individual variation in lung tissue behavior.

Although different lung lobes are known to exhibit varying motion amplitudes, our analysis did not reveal consistent differences in motion error across lobes. This may be due to the small and uneven sample sizes per lobe (3–8 patients), limiting the statistical power of lobe‐specific comparisons. Instead, we observed a negative correlation between the D‐vDSC of lung tumors and TCM motion errors. Specifically, higher tumor deformation (indicated by lower D‐vDSC values) was associated with larger TCM motion errors. This suggests that increased tumor deformation complicates tumor contour alignment during image registration, leading to higher motion errors. This finding is critical for ensuring the accuracy of radiation therapy planning. Future studies should evaluate the model across a broader range of patient datasets to enhance its robustness and clinical applicability, particularly for real‐time tumor tracking during treatment. For example, subjects *S*_1_ and *S*_13_, who exhibited the smallest D‐vDSC values of 0.74 and 0.77, also demonstrated the largest TCM motion errors of 3.60 mm and 3.42 mm, respectively. This may indicate more complex tumor deformation in these subjects, potentially due to heterogeneous tissue properties or significant nonlinear motion during the breathing cycle.

### 4.2 Lung surface traction forces and tumor motion

The lung STVFs were identified as critical factors driving lung tumor motion. A strong positive correlation (*r* > 0.82) was observed between tumor motion amplitude and STVFs, particularly in region where the extension of the TCM intersected with STVFs on the lung between the end‐expiration and end‐inspiration phases. This relationship likely indicates that lung deformation mechanically drives the directional trend of tumor displacement. Using deformation‐based image registration, surface displacement vector fields (SDVFs) were derived and subsequently converted into STVFs through patient‐specific elasticity parameters. These STVFs quantified the forces influencing lung deformation and tumor motion, providing a novel biomechanical perspective that links lung deformation to tumor motion. Although STVFs are not directly measurable in clinical practice, they offer a physically interpretable correlate of lung deformation effects on tumor motion. This understanding may enhance motion modeling frameworks and support future development of deformation‐informed treatment adaptation.

Although the FFD‐based registration provided clinically validated displacement fields, uncertainties in 4DCT acquisition and registration accuracy may propagate through the biomechanical workflow and affect the optimized elasticity parameters. These uncertainties were not explicitly modeled in this study but are recognized as important factors. Future work will incorporate uncertainty modeling into the optimization framework to improve the robustness and interpretability of the estimated mechanical properties.

### 4.3 Optimized elasticity parameters and model accuracy

Few studies have explored the dynamics of lung elasticity parameters in detail. To our knowledge, only one study has used a quadratic function to model the Poisson's ratio during the inspiratory phases.^36^ The optimized elasticity parameters, fitted using a first‐order Fourier function, showed excellent fitting performance across the entire respiratory cycle, with R^2^ values exceeding 0.85 for all patients (Figure 3(a)). This high accuracy likely results from the periodic nature of lung motion during respiration, which can be approximated as a periodic waveform. The first‐order Fourier function captures the dominant oscillatory behavior of lung motion, making it ideal for modeling elasticity parameters variations across respiratory phases. This approach offers new insights into the dynamic evolution of lung elasticity, contributing to a deeper understanding of respiratory mechanics.

The optimization of elasticity parameters was informed by tumor motion and deformation observed in CT images. Previous studies have demonstrated a close relationship between CT images and tissue physical properties such as density^37^ and elasticity.^38^ For example, 4DCT imaging reveals significant variations in the Poisson's ratio of the lung due to changes in alveolar volume and air content during the respiratory cycle.^16^ This method allows for precise extraction of elasticity parameters, which can be integrated into biomechanical models to predict tumor motion and deformation with high accuracy.

In our LASSO regression analysis, only age was found to significantly affect both Young's modulus and Poisson's ratio. These findings are consistent with those of S. J. Lai‐Fook et al.^39^ who observed that aging is associated with decreased Young's modulus and an increased in Poisson's ratio. This is likely the fact that as age increases, lung elasticity diminishes, and the relative resistance to uniform expansion (bulk modulus) increases compared to shear resistance (shear modulus). Additionally, features such as *X*_1_, *X*_2,_ and *X*_11_ were positively correlated with higher Young's modulus. This is because greater relative volume changes (*X*_1_) indicate higher ventilation capacity, a larger relative change in the average CT value (*X*_2_) reflects higher lung density, and a larger Patient's Slab Rod Index (*X*_11_) suggests greater lung deformation. And a larger body mass index (*X*_9_) correlates with a higher Poisson's ratio, likely due to increased fat content and reduced lung compliance, which enhances lateral deformation under compression. These results highlight the importance of individualized models in enhancing the accuracy of biomechanical simulations, ultimately leading to more effective and personalized medical interventions. Although the direct clinical estimation of lung elasticity remains challenging, the identified correlations between patient characteristics and optimized parameters suggest that surrogate models could eventually estimate biomechanical properties from routinely available clinical data, potentially reducing the need for complex image registration procedures.

### 4.4 Limitations and future directions

The biomechanical model may serve as a simulation tool to better understand tumor motion and deformation under different breathing conditions, which is essential for designing personalized motion management strategies in radiotherapy. However, this study has several important limitations.

First, the data used sourced from a single center, comprising 27 cases with slice thicknesses of 2 mm (4 cases) and 3 mm (23 cases). The limited number of cases with 2 mm slices restricts the generalizability of the findings. However, as a proof‐of‐concept study, the sample size is relatively substantial and serves to demonstrate the initial feasibility of the proposed method. Future studies should incorporate multi‐center data and a more diverse patient cohort, including varying slice thicknesses, to improve the accuracy and applicability of LTMD simulations.

Second, the SKV and NH models were selected because they explicitly utilize Young's modulus and Poisson's ratio, which are pivotal parameters in our study. These parameters are of central importance in our investigation, and both models have been widely applied in the field of lung biomechanics. Within the deformation range of our dataset, which was consistently below 40%, these models effectively approximate the strain behavior of the lungs. However, their accuracy may diminish when dealing with large deformations or attempting to capture intricate nonlinear material behavior. For future research, we intend to integrate more sophisticated hyperelastic models, such as the Ogden or Mooney‐Rivlin formulations. By doing so, we aim to enhance the physiological accuracy of lung biomechanical simulations, especially under more extreme respiratory conditions.

Third, this study focused on accurately predicting tumor motion and deformation; therefore, the objective function was defined solely within the tumor region. This approach is supported by previous study.^16^ Future work may incorporate additional constraints, such as lung deformation errors or stress field consistency, into a composite objective function. Moreover, while this study optimized overall lung elasticity parameters from 4DCT images, it did not account for their spatial heterogeneity. Previous studies have showed a linear correlation between lung elasticity modulus and CT values,^38^ suggesting that future studies should address the spatial variation in elasticity parameters for more precise modeling of both lung and tumor biomechanical behavior.

Moreover, although this study focused on respiration‐induced tumor motion, the combined effects of respiratory and cardiac‐induced tumor motion may increase model complexity. To broaden the model's applicability, future research should integrate both respiratory and cardiac influences on LTMD. Additionally, personalized models tailored to specific patient populations, such as those with Chronic Obstructive Pulmonary Disease (COPD), should be explored.

All simulations in this study were conducted under the assumption of supine positioning, consistent with standard 4DCT acquisition protocols. The influence of gravity and posture on lung deformation and traction distribution was therefore not explicitly modeled. Finally, the construction of personalized biomechanical models remains time‐consuming, involving geometry creation, parameter optimization, image registration, and FEM. The integration of artificial intelligence with advanced techniques like radiomics or delta‐radiomics could streamline this process by enabling rapid estimation of lung elasticity parameters from 4DCT data. This would facilitate real‐time guidance for lung motion management while continuously improving model accuracy through iterative training.

## 5 CONCLUSION

This study presents the development of a time‐varying, bi‐parametric hyper‐elastic biomechanical model using the FEAIR method, demonstrating its potential for accurately estimating lung tumor motion and deformation. The proposed framework emphasizes the importance of personalized and dynamic modeling in improving predictive accuracy. Future research should focus on increasing the sample size and incorporating dynamic lung mechanics to further enhance the model's robustness and clinical applicability.

## CONFLICT OF INTEREST STATEMENT

The authors declare no conflicts of interest.

## Supporting information



Supporting information

## Data Availability

The imaging data contains patient privacy information. However, reasonable requests for access will be considered and can be provided upon request.
