# Supplementary material for: A novel personalized time‐varying biomechanical model for estimating lung tumor motion and deformation
Source: Med Phys. 2025 Sep 3;52(8):e18086. doi: 10.1002/mp.18086 (PMC12409114; doi:10.1002/mp.18086)
Supplement: Supplementary file 1 — Supporting information [file MP-52-0-s001.docx]

**Table S1.** Quantitative comparison of volume (V, cm^3^) and surface area (S, cm^2^) between segmentation masks (in NIfTI format) and stereolithography (STL) models across respiratory phases.

| Phase | Lung V (Mask) | Lung V (STL) | Lung S (Mask) | Lung S (STL) | Tumor V (Mask) | Tumor V (STL) | Tumor S (Mask) | Tumor S (STL) |
| --- | --- | --- | --- | --- | --- | --- | --- | --- |
| P00 | 2077.44 | 2055.88 | 1021.32 | 1020.55 | 10.09 | 10.31 | 24.88 | 25.00 |
| P10 | 2036.85 | 2016.04 | 999.52 | 997.90 | 4.28 | 4.26 | 28.25 | 28.42 |
| P20 | 2022.37 | 2013.71 | 986.64 | 979.31 | 7.65 | 7.58 | 25.28 | 25.51 |
| P30 | 1962.34 | 2016.86 | 968.49 | 980.05 | 11.76 | 11.80 | 33.44 | 33.88 |
| P40 | 1917.70 | 1898.35 | 977.13 | 961.66 | 10.63 | 10.42 | 25.85 | 25.51 |
| P50 | 1904.12 | 1893.06 | 968.34 | 963.26 | 6.43 | 6.18 | 20.82 | 21.12 |
| P60 | 1928.18 | 1939.41 | 1022.90 | 1031.98 | 10.17 | 10.14 | 17.32 | 17.16 |
| P70 | 1974.91 | 1952.95 | 965.60 | 968.68 | 4.46 | 4.48 | 29.16 | 29.60 |
| P80 | 2018.48 | 2020.95 | 967.05 | 962.07 | 4.52 | 4.48 | 16.95 | 17.19 |
| P90 | 2072.62 | 2041.36 | 956.36 | 966.87 | 6.49 | 6.60 | 27.75 | 27.38 |

**Table S2.** Fitting accuracy (R² and MSE) of low-complexity models (Exp2, Fourier1, Gauss2, Poly2) for Young’s modulus evolution across respiratory phases.

| Cases | Exp2 | | Fourier1 | | Gauss2 | | Poly2 | |
| --- | --- | --- | --- | --- | --- | --- | --- | --- |
|  | R2 | MSE | R2 | MSE | R2 | MSE | R2 | MSE |
| case1 | 0.8242 | 0.0013 | 0.9112 | 0.0012 | 0.8789 | 0.0005 | 0.7737 | 0.0017 |
| case2 | 0.8496 | 0.0012 | 0.9045 | 0.0004 | 0.8684 | 0.0006 | 0.8279 | 0.0013 |
| case3 | 0.9365 | 0.0004 | 0.9357 | 0.0003 | 0.9243 | 0.0002 | 0.9336 | 0.0005 |
| case4 | 0.8833 | 0.0002 | 0.8896 | 0.0005 | 0.8551 | 0.0001 | 0.7637 | 0.0003 |
| case5 | 0.9987 | 0.0000 | 0.9315 | 0.0003 | 0.9177 | 0.0007 | 0.9313 | 0.0014 |
| case6 | 0.8773 | 0.0010 | 0.8911 | 0.0002 | 0.8423 | 0.0008 | 0.8133 | 0.0015 |
| case7 | 0.9159 | 0.0003 | 0.9231 | 0.0001 | 0.8802 | 0.0002 | 0.9150 | 0.0003 |
| case8 | 0.8632 | 0.0009 | 0.9399 | 0.0005 | 0.8326 | 0.0048 | 0.7664 | 0.0016 |
| case9 | 0.8894 | 0.0004 | 0.9413 | 0.0001 | 0.8984 | 0.0002 | 0.8435 | 0.0006 |
| case10 | 0.8287 | 0.0014 | 0.9390 | 0.0008 | 0.9094 | 0.0003 | 0.7860 | 0.0018 |
| case11 | 0.9751 | 0.0001 | 0.9587 | 0.0006 | 0.9261 | 0.0001 | 0.9195 | 0.0003 |
| case12 | 0.8286 | 0.0008 | 0.9147 | 0.0004 | 0.8786 | 0.0003 | 0.7624 | 0.0011 |
| case13 | 0.9612 | 0.0001 | 0.9475 | 0.0005 | 0.8216 | 0.0001 | 0.9410 | 0.0001 |
| case14 | 0.8785 | 0.0012 | 0.9895 | 0.0002 | 0.8248 | 0.0080 | 0.8300 | 0.0016 |
| case15 | 0.8980 | 0.0003 | 0.8652 | 0.0004 | 0.9182 | 0.0001 | 0.8666 | 0.0005 |
| case16 | 0.8652 | 0.0012 | 0.9514 | 0.0007 | 0.9083 | 0.0004 | 0.8640 | 0.0012 |
| case17 | 0.9733 | 0.0001 | 0.8904 | 0.0003 | 0.9233 | 0.0001 | 0.9316 | 0.0004 |
| case18 | 0.9032 | 0.0011 | 0.9456 | 0.0015 | 0.8793 | 0.0008 | 0.8697 | 0.0015 |
| case19 | 0.9067 | 0.0004 | 0.9288 | 0.0004 | 0.8578 | 0.0004 | 0.8982 | 0.0005 |
| case20 | 0.8620 | 0.0006 | 0.9068 | 0.0004 | 0.8880 | 0.0003 | 0.7995 | 0.0009 |
| case21 | 0.8431 | 0.0009 | 0.9366 | 0.0004 | 0.9213 | 0.0002 | 0.8254 | 0.0010 |
| case22 | 0.8534 | 0.0004 | 0.9176 | 0.0008 | 0.8504 | 0.0003 | 0.8031 | 0.0005 |
| case23 | 0.8923 | 0.0011 | 0.9064 | 0.0002 | 0.9223 | 0.0003 | 0.8919 | 0.0011 |
| case24 | 0.8110 | 0.0014 | 0.9179 | 0.0002 | 0.9314 | 0.0001 | 0.8144 | 0.0014 |
| case25 | 0.9169 | 0.0007 | 0.8797 | 0.0003 | 0.8918 | 0.0005 | 0.8156 | 0.0016 |
| case26 | 0.8797 | 0.0006 | 0.9370 | 0.0001 | 0.9202 | 0.0002 | 0.7370 | 0.0014 |
| case27 | 0.8665 | 0.0007 | 0.9753 | 0.0007 | 0.7356 | 0.0001 | 0.7665 | 0.0016 |

**Table S3.** Fitting accuracy (R² and MSE) of low-complexity models (Exp2, Fourier1, Gauss2, Poly2) for Poisson’s ratio evolution across respiratory phases.

| Cases | Exp2 | | Fourier1 | | Gauss2 | | Poly2 | |
| --- | --- | --- | --- | --- | --- | --- | --- | --- |
|  | R2 | MSE | R2 | MSE | R2 | MSE | R2 | MSE |
| case1 | 0.7715 | 0.0026 | 0.8972 | 0.0007 | 0.8266 | 0.0014 | 0.7740 | 0.0026 |
| case2 | 0.9412 | 0.0004 | 0.9453 | 0.0007 | 0.9155 | 0.0002 | 0.9411 | 0.0004 |
| case3 | 0.8701 | 0.0003 | 0.8799 | 0.0004 | 0.8410 | 0.0003 | 0.8538 | 0.0003 |
| case4 | 0.8903 | 0.0004 | 0.8728 | 0.0001 | 0.8570 | 0.0003 | 0.7797 | 0.0008 |
| case5 | 0.8513 | 0.0005 | 0.9148 | 0.0014 | 0.8789 | 0.0002 | 0.5746 | 0.0015 |
| case6 | 0.8897 | 0.0002 | 0.9078 | 0.0008 | 0.8491 | 0.0002 | 0.8642 | 0.0003 |
| case7 | 0.9407 | 0.0001 | 0.9452 | 0.0003 | 0.9048 | 0.0001 | 0.9406 | 0.0001 |
| case8 | 0.9246 | 0.0005 | 0.9230 | 0.0004 | 0.8761 | 0.0005 | 0.9122 | 0.0006 |
| case9 | 0.9170 | 0.0001 | 0.9400 | 0.0002 | 0.9090 | 0.0000 | 0.9044 | 0.0001 |
| case10 | 0.8661 | 0.0009 | 0.8852 | 0.0005 | 0.8253 | 0.0008 | 0.8560 | 0.0009 |
| case11 | 0.7693 | 0.0009 | 0.8563 | 0.0001 | 0.7737 | 0.0007 | 0.7149 | 0.0011 |
| case12 | 0.9332 | 0.0005 | 0.9491 | 0.0004 | 0.8956 | 0.0004 | 0.9310 | 0.0005 |
| case13 | 0.9063 | 0.0003 | 0.8494 | 0.0001 | 0.8942 | 0.0002 | 0.6038 | 0.0011 |
| case14 | 0.9456 | 0.0002 | 0.9397 | 0.0001 | 0.9181 | 0.0001 | 0.9396 | 0.0002 |
| case15 | 0.8561 | 0.0006 | 0.9030 | 0.0013 | 0.8285 | 0.0005 | 0.8357 | 0.0006 |
| case16 | 0.8963 | 0.0009 | 0.9234 | 0.0002 | 0.8890 | 0.0005 | 0.8929 | 0.0009 |
| case17 | 0.7937 | 0.0007 | 0.9356 | 0.0010 | 0.8507 | 0.0004 | 0.7238 | 0.0010 |
| case18 | 0.8693 | 0.0022 | 0.9119 | 0.0003 | 0.8510 | 0.0017 | 0.8647 | 0.0023 |
| case19 | 0.7994 | 0.0006 | 0.8642 | 0.0008 | 0.7534 | 0.0006 | 0.7986 | 0.0006 |
| case20 | 0.8147 | 0.0009 | 0.9139 | 0.0004 | 0.8351 | 0.0005 | 0.7065 | 0.0014 |
| case21 | 0.8470 | 0.0005 | 0.8919 | 0.0003 | 0.8890 | 0.0002 | 0.5775 | 0.0013 |
| case22 | 0.8850 | 0.0016 | 0.9472 | 0.0005 | 0.9026 | 0.0007 | 0.8576 | 0.0020 |
| case23 | 0.9446 | 0.0001 | 0.9209 | 0.0002 | 0.9033 | 0.0001 | 0.7889 | 0.0004 |
| case24 | 0.8892 | 0.0002 | 0.9086 | 0.0009 | 0.8866 | 0.0001 | 0.8826 | 0.0003 |
| case25 | 0.8381 | 0.0003 | 0.8495 | 0.0009 | 0.7954 | 0.0003 | 0.8292 | 0.0003 |
| case26 | 0.9382 | 0.0001 | 0.9543 | 0.0006 | 0.9128 | 0.0001 | 0.7617 | 0.0006 |
| case27 | 0.8715 | 0.0008 | 0.8842 | 0.0001 | 0.8280 | 0.0007 | 0.8579 | 0.0008 |

**Table S4.** True tumor motion amplitudes and corresponding prediction errors across 27 patients.

| Patients | True Motion  Amplitude (mm) | Prediction  Error (mm) |
| --- | --- | --- |
| case1 | 0.50 | 0.41 |
| case2 | 0.73 | 0.48 |
| case3 | 0.84 | 0.62 |
| case4 | 0.94 | 0.68 |
| case5 | 0.96 | 0.62 |
| case6 | 1.55 | 0.72 |
| case7 | 1.86 | 1.12 |
| case8 | 2.04 | 1.69 |
| case9 | 2.31 | 1.54 |
| case10 | 2.62 | 1.22 |
| case11 | 2.73 | 1.70 |
| case12 | 2.77 | 1.41 |
| case13 | 3.50 | 1.29 |
| case14 | 3.62 | 1.41 |
| case15 | 3.92 | 2.42 |
| case16 | 5.47 | 1.75 |
| case17 | 7.63 | 1.50 |
| case18 | 8.14 | 1.65 |
| case19 | 9.10 | 1.61 |
| case20 | 10.92 | 1.82 |
| case21 | 11.21 | 1.15 |
| case22 | 12.46 | 1.98 |
| case23 | 12.46 | 1.88 |
| case24 | 13.22 | 1.26 |
| case25 | 14.54 | 2.18 |
| case26 | 15.23 | 2.15 |
| case27 | 17.88 | 3.60 |


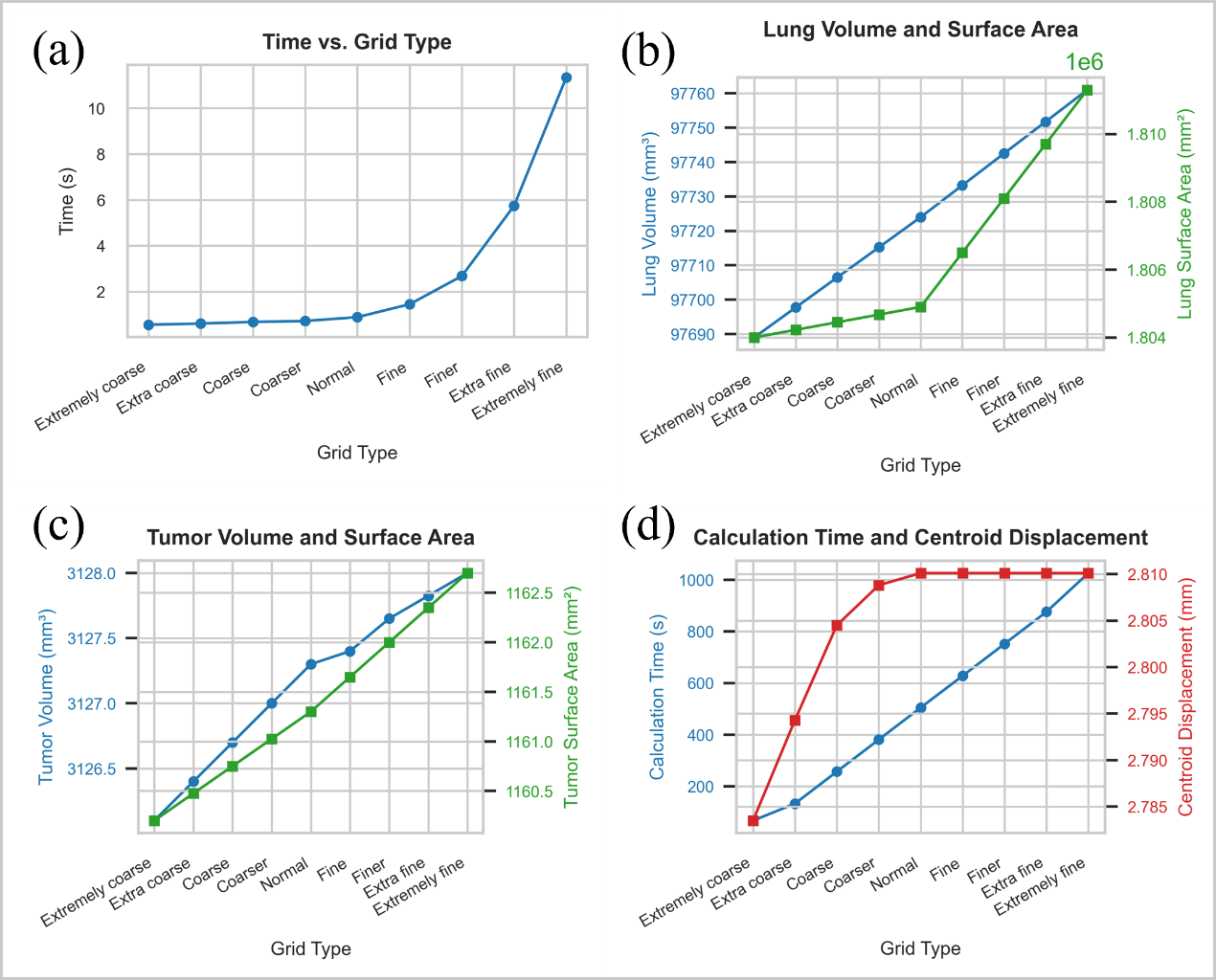


**Figure S1.** A representative evaluation of mesh resolution effects on geometry construction accuracy and computational stability. It presents the influence of COMSOL-generated tetrahedral mesh resolution on geometry import time, anatomical fidelity, and biomechanical simulation stability. Mesh types ranged from "Extremely coarse" to "Extremely fine", automatically adapted to geometric complexity. (a) Geometry construction time vs. mesh resolution. (b) Lung volume (blue) and surface area (green). (c) Tumor volume (blue) and surface area (green). (d) Mesh convergence results. Tumor centroid displacement stabilizes from the Normal mesh upward, while calculation time increases with finer meshes.


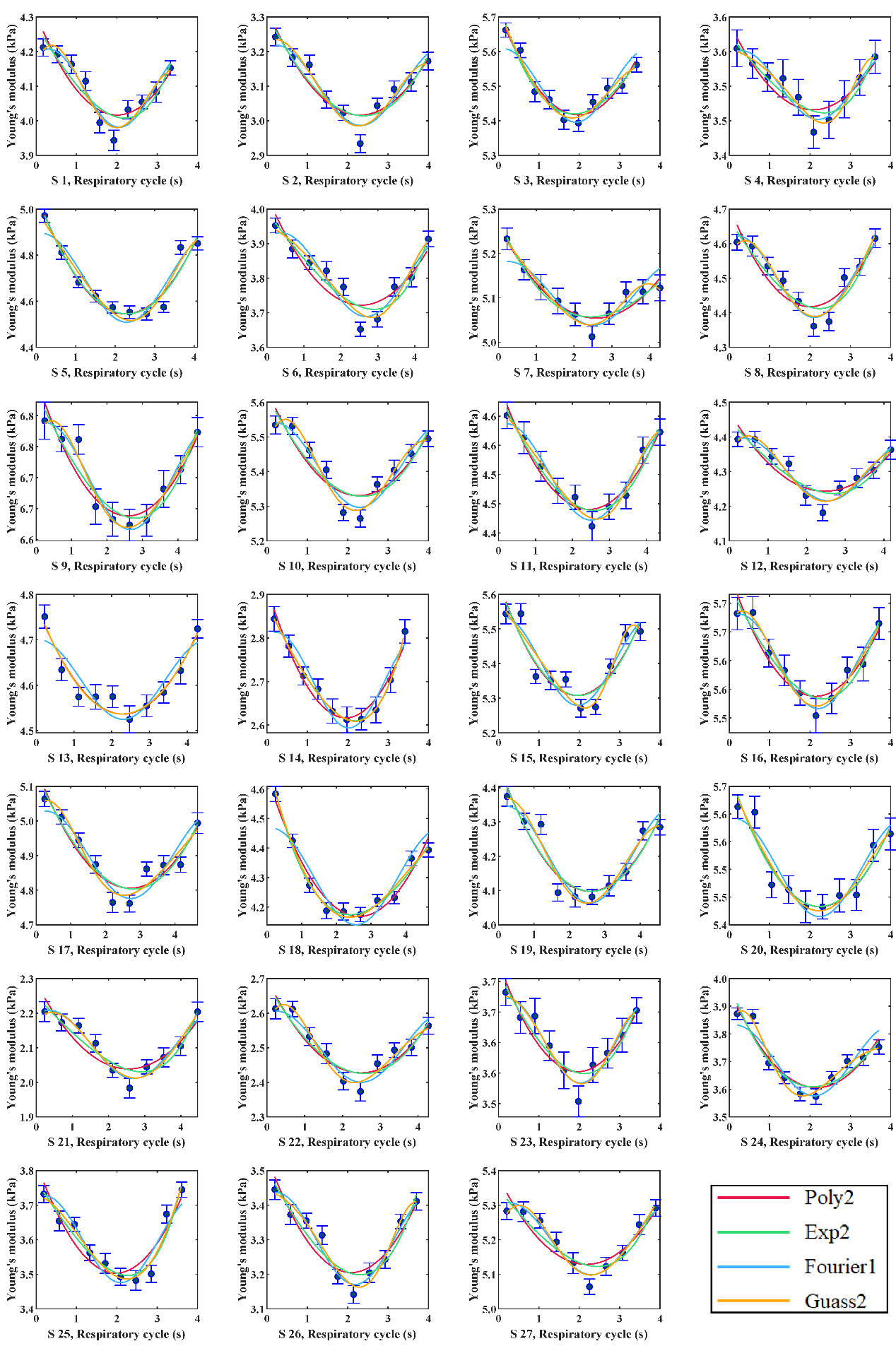


**Figure S2.** Model fitting of personalized lung Young’s modulus across the respiratory cycle in 27 subjects.

**
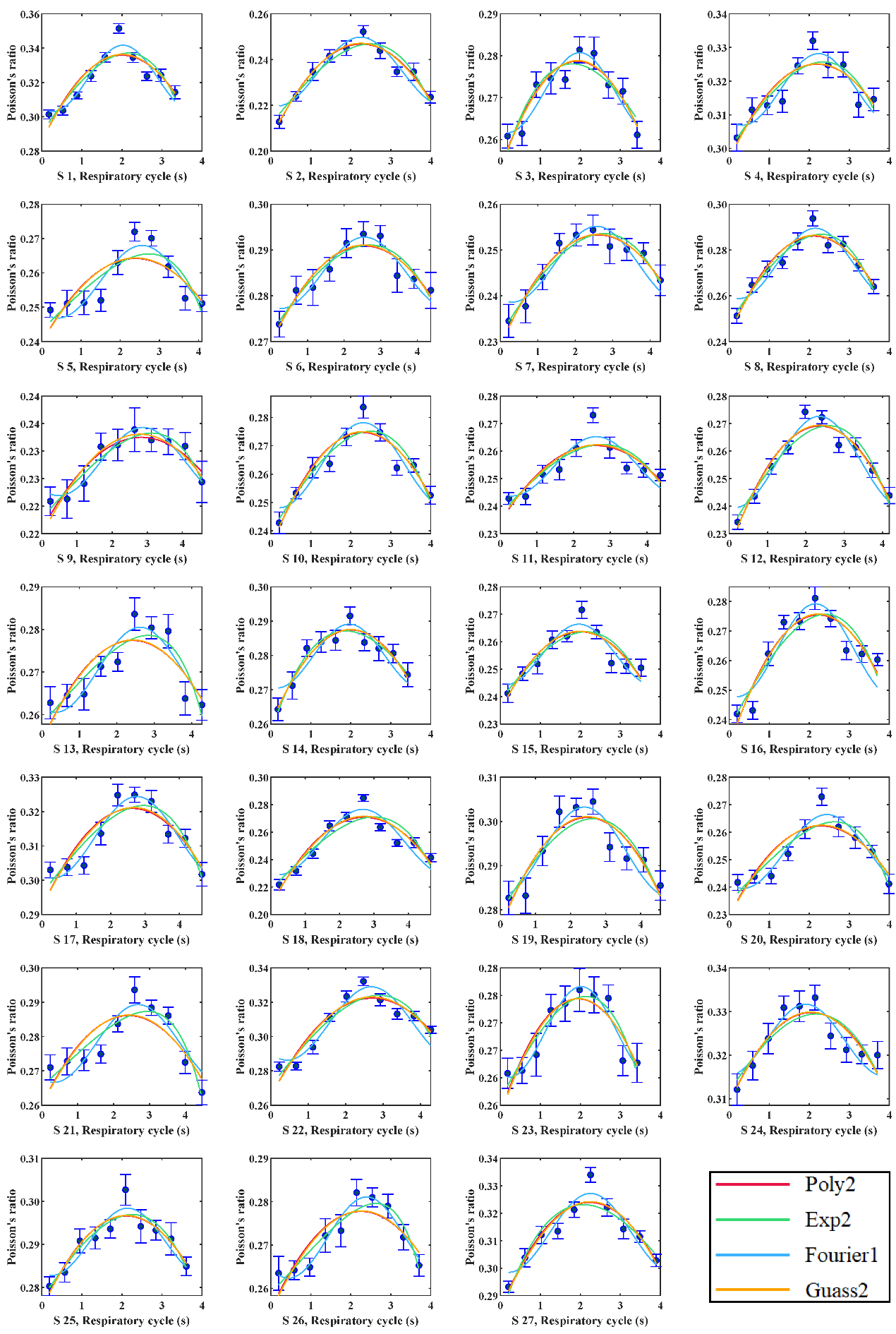
**

**Figure S3.** Model fitting of personalized lung Poisson’s ratio across the respiratory cycle in 27 subjects.
